# Preliminary Data From the Study of Coagulative Profile of HIV Infected Individuals Suggest a Role For Point Mutations in the Gene in Protein S Deficiency in Individuals Undergoing Highly Antiretroviral Therapy

**DOI:** 10.2174/1874613601812010006

**Published:** 2018-02-28

**Authors:** Mariantonietta Di Stefano, Giovanna D’Andrea, Fabio Zoboli, Giuseppina Faleo, Massimo Fasano, Domenico Martinelli, Maurizio Margaglione, Teresa A. Santantonio, Josè R. Fiore

**Affiliations:** 1 Department of Clinical and Experimental Medicine, Section of Infectious Diseases, University of Foggia, Viale L. Pinto 71131 Foggia, Italy; 2 Medical Genetics, University of Foggia, Viale L. Pinto 71131 Foggia, Italy; 3 Department of Medical Sciences, Section of Hygiene, University of Foggia, Apulia Regional Epidemiological Observatory, Viale L. Pinto 71131 Foggia, Italy

**Keywords:** Protein S, Protein C, Thrombosis, HIV, Antiretrovirals, Pros 1 gene, PS deficiency

## Abstract

**Background:**

HIV infection is a known prothrombotic condition but factors involved are still controversial. A role for antiretrovirals, especially protease inhibitors, was advocated.

**Objectives:**

The study aimed to analyze the levels of anticoagulant proteins in virally suppressed HIV-infected subjects treated with different anti-retroviral regimens.

**Materials and Methods:**

Forty-four patients were included in the study. C and PS, D-Dimers and Fibrinogen levels were determined as well as APC-resistance. PROS1 gene was sequenced in a group of patient.

**Results:**

Twelve of the 44 subjects (27%) showed reduced levels of PS, while lower levels of PC were found only in 2 patients (4,5%). No difference in the mean values of PC and PS was found stratifying the study population by antiretroviral regimen administrated (p>0.05).

Three patients had higher levels of D-Dimer concentrations and in two of these patients, an association between higher D-Dimer values and lower levels of PS was observed; but however no correlation was found by statistical analysis.

PROS1 gene analysis was performed in 26 of the 44 HIV-1 patients and the subjects with low levels of PS had mutation in the fifteen exon of PROS 1 gene. While among individuals with normal levels, this mutation was observed only in 8/18 (44%) of the cases (p=0,0072).

**Conclusion:**

The majority of patients with low PS levels also had mutations in the fifteen exon of PROS 1 gene. Genetic determinants, deserving further investigations, rather than antiretrovirals might cause PS deficiency in HIV-1 positive patients.

## 1 INTRODUCTION

HIV infection is recognized as possibly responsible for abnormal coagulative profile leading to increased risk of thromboembolic events [1-4]. A low CD4 cell count was demonstrated a strong predictor of thrombotic events [5] as well as the stage of the disease [6] and the presence of opportunistic infections and malignancies [7].

In addition, high viral burden was associated with thrombotic events in one study [8] but this result was not confirmed and is still controversial [7].

Regarding antiretrovirals, HAART treatment, especially protease inhibitor containing regimens, has been associated with thrombotic events, possibly because of the interference with the regulation of thrombotic proteins leading to increased fibrinogen, D dimer, plasminogen activator inhibitor 1 or protein S deficiency [9]. However, also these results, not confirmed in other studies [8], are still a matter of debate.

Here we present the preliminary results from an ongoing study on HIV infected patients treated with different HAART regimens and virally suppressed, aimed to better define the possible prothrombotic determinants during antiretroviral treatments.

## 2 PATIENTS, MATERIAL AND METHODS

Forty-four consecutive patients referring to the infectious diseases outpatient section at the University Hospital of Foggia, (Foggia, Italy) were enrolled according to the following inclusion criteria: 1) to be diagnosed as HIV infected 2) to be at least of 18 years and capable of properly understanding the purposes of the study and sign an informed consent 3) be apparently healthy at clinician screening and with no history of opportunistic infections or neoplasm in the last six months 4) no known history of coagulation or bleeding disorders or coagulation-affecting therapies in the last six months 5) to be a nonsmoker from at least six months 6) no history of diabetes, high blood pressure, increased cholesterol in blood 7) to be under antiretroviral treatment and virally suppressed in plasma from at least 6 months.

HIV-1 and HCV RNA were quantified by a commercially available real time assay (ABBOTT GmbH & Co KG Max Planck-Ring 2 65205 Wiesbaden Germany). Detection limits were 75 copies/ml for HIV and 12 UI/ml for HCV,

C and S protein were quantified in serum or plasma by using a commercially available chromogenic assay. (Instrumentation Laboratory Company Bedford - MA 01730-2443 USA). Normal ranges for PC and PS were 70 to 140 IU/mL and 50 to 130, respectively.

A functional activated protein C resistance test was used to determine a Factor V related APC ratio (APC-V ratio) determining the presence of Activated Protein C resistance (APCr). For this purpose, a commercial assay kit (APC Resistance V 823120, Chromogenix Instrumentation Laboratory Company, Lexington MA USA) was used.

D-Dimers were measured in plasma by an automated latex enhanced immunoassay (Instrumentation Laboratory Company Bedford - MA 01730-2443 USA). For the quantitative determination of D-Dimer in plasma, a value was defined normal below 250 ng/mL .

Fibrinogen was measured by a commercially available assay (Instrumentation Laboratory company Bedford-MA, USA) with normal values ranging from 200 to 450 mg/dl.

A mutation in the PROS1 gene, located in chromosome 3 at position 3p 11.1, was analyzed by Polymerase Chain Reaction (PCR) and sequencing [10] on DNA extracted from peripheral blood. The sequencing of the amplified product was performed using an automated DNA sequencing analyzer (Applied Bio systems, Foster City, CA, USA), according to the manufacturer's instruction.

The obtained data were analyzed stratifying the study population by antiretroviral regimen administrated. The difference in the mean values of PC and PS according to antiretroviral regimen administrated was investigated by the t-test, considering significant *p*-values <0,05.

## 3 RESULTS

The patient series included 44 patients (32 HIV mono-infected; 10 HIV/HCV co-infected and 2 HIV/HBV coinfected) ten were females and 34 were males. The mean age was 48 years (range from 28 to 69), while CD4+ cell count was 674 (range from 119 to 1193/mm^3^)

The mean D-Dimer concentration of the patients was 110,7 ug/ml (range from 18 to 317). Three patients had higher levels of D-Dimer concentrations and in two of three patients an association with lower levels of PS was observed, though non-significant. Three patients had fibrinogen levels below the normal range from 200 to 450, with no significant correlation observed.

Regarding PS and PC levels, reduced levels of S protein were observed in 12/44 (27%) patients, from 29 to 49 IU/ml. Low levels of PC were found only in 2 patients (4,5%). The remaining patients showed normal levels of both anticoagulant markers.

No correlation was found between levels of anticoagulant markers and the type of antiretroviral regimen administrered. In addition, no correlation was observed with CD4 cell counts, HCV and HBV co-infections.

No difference in PC and PS concentrations was found stratifying the study population by antiretroviral regimen administrated (p>0.05).

PROS1 gene analysis was performed in 8 HIV-1 patients with low and 18 Interestingly enough, in six patients an A->G at nucleotide 2148 (also called P626) of PROS1 gene was found whereas two individuals resulted homozygous for the PROS1 gene showing a GG genotype for the nucleotide 2148 in with normal S protein level (Table **1**).

An A->G nucleotide substitution at position 2148 (also called P626) of PROS1 gene was found in 16 individuals and namely in all of the subjects with low PS levels and in 8 out of 18 subjects with normal PS levels (p-value 0.0072). Homozygosity occurred in 3 of 8 patients with low and in 2 out of 18 patients with normal levels of PS. Among eighteen HIV-1 patients with normal levels of PS, three patients were homozygous for the P626 having a GG genotype, five were heterozygous (AG genotype), the remaining had a wild type genotype (AA genotype).

The correlation between low S protein levels and mutation in the PROS1 gene was statistically significant (p: 0.0072)

## 4 DISCUSSION

HIV infection is a known prothrombotic condition but factors involved are still controversial.

Several co-factors (such as viral load, CD4 cell counts, opportunistic infections, malignancies and others) have been proposed, at a certain extent, as co-factors in the phenomenon.

Also HAART treatment, especially protease inhibitor containing regimens, has been proposed [9]. However, these results were not confirmed from other studies [8].

Here we present preliminary results from an ongoing study on HIV infected patients treated with different HAART regimens and virally suppressed, aimed to better define the possible prothrombotic determinants during antiretroviral treatments. The results highlight the possible role played in this phenomenon by mutations in the gene involved in PS modulation.

PC and PS are natural anticoagulants of key importance in haemostasis regulation and thrombosis. In fact, cohort studies demonstrated increased risk of Venous Thromboembolism (VTE) in individuals with PC and/or PS deficiency and family history of VTE episodes [6]. In addition, it is known that PS deficiency can be caused by mutation in the PROS1 gene in fact, patients with mutation at position 2148 of PROS 1 (also termed P626) may have low levels of (dysfunctional) PS leading to an increased risk for abnormal blood clots [10].

In this study PROS1 gene analysis was performed in patients with low and normal PS levels. Interestingly enough, an A->G nucleotide substitution at position 2148 (also called P626) of PROS1 gene was found in 16 individuals and namely in all of the subjects with low PS levels and in 44% of subjects with normal PS levels (*P*-value 0.0072). In addition, homozygosity occurred in 37% of patients with low and in 11% of patients with normal levels of PS. This appeared unrelated to the type of antiretroviral therapy administered.

In a recent study, [11, 12] a correlation was observed between HIV positive serostatus and protein S deficiency in Zambia. Our study add possibly valuable information to these results. In fact, although limited by the small size of patients studied, this is, at our knowledge, the first report evaluating the correlation between protein S levels and gene mutations among HAART treated HIV infected individuals. In spite of this limitation results presented here strongly suggests that genetic determinants rather may play a predominant role in PS deficiency in HAART treated HIV-1 positive patients.

In spite of the clear correlation between the detection of the mutation investigated and the decrease in PS levels, not all of the patients carrying it had low levels. Several genetic factors are involved in PS modulation [13]. Further investigations are needed to individuate others co-factors possibly involved, the real size of the phenomenon and whether a screening assessment of genetic coagulative characteristics of HIV infected patients might be helpful as a predictive marker of thrombotic events in these patients.

## CONCLUSION

In the search for factors involved in prothrombotic conditions in HAART treated HIV infected patients, we present here preliminary data on coagulative profile of individuals treated with different HAART regimens.

The levels of S protein in the studied patients appeared not related to the type of drug combination administered: a statistically significant correlation, instead, was observed with the presence of a mutation in the position 2148 (P626) of the PROS1 gene.

Although based on a small patient series, these results suggest that genetic factor may act, at least as important cofactors, in S protein levels reduction observed in HAART treated individuals.

Further studies on larger cohorts are needed to understand the real size of the phenomenon.

## Figures and Tables

**Table 1:** Levels of S protein in 26 patients according to PROS 1 gene mutations.

S protein levels		
	low*	normal*
No mutation	0	10
Heterozygousis	5	6
Homozygousis	3	2

*Levels of Protein S in blood were considered normal if higher than 50 IU/ml.

## AVAILABILITY OF DATA AND MATERIALS

The data supporting these findings will not be made available publically due to concerns about protecting participants’ identity and respecting their rights to privacy. At the time the study was conducted, we did not obtain informed consent from participants for publication of the dataset.
